# *cis *sequence effects on gene expression

**DOI:** 10.1186/1471-2164-8-296

**Published:** 2007-08-29

**Authors:** Andrew W Bergen, Andrea Baccarelli, Timothy K McDaniel, Kenneth Kuhn, Ruth Pfeiffer, Jerry Kakol, Patrick Bender, Kevin Jacobs, Bernice Packer, Stephen J Chanock, Meredith Yeager

**Affiliations:** 1 Division of Cancer Epidemiology and Genetics, National Cancer Institute, National Institutes of Health, Bethesda, MD USA; 2 Center for Health Sciences, Policy Division, SRI International, Menlo Park, CA USA; 3 School of Public Health, Harvard University, Boston, MA USA; 4 Molecular Epidemiology and Genetics, EPOCA Epidemiology Center, Maggiore Hospital, Mangiagalli and Regina Elena IRCCS Foundation & University of Milan, Milan, Italy; 5 Illumina, San Diego, CA USA; 6 Coriell Institute for Medical Research, Camden, NJ USA; 7 Core Genotyping Facility, National Cancer Institute, Gaithersburg, MD USA; 8 Science Applications International Corporation-National Cancer Institute (NCI), NCI-FCRDC, Frederick, MD USA

## Abstract

**Background:**

Sequence and transcriptional variability within and between individuals are typically studied independently. The joint analysis of sequence and gene expression variation (genetical genomics) provides insight into the role of linked sequence variation in the regulation of gene expression. We investigated the role of sequence variation in *cis *on gene expression (*cis *sequence effects) in a group of genes commonly studied in cancer research in lymphoblastoid cell lines. We estimated the proportion of genes exhibiting *cis *sequence effects and the proportion of gene expression variation explained by *cis *sequence effects using three different analytical approaches, and compared our results to the literature.

**Results:**

We generated gene expression profiling data at N = 697 candidate genes from N = 30 lymphoblastoid cell lines for this study and used available candidate gene resequencing data at N = 552 candidate genes to identify N = 30 candidate genes with sufficient variance in both datasets for the investigation of *cis *sequence effects. We used two additive models and the haplotype phylogeny scanning approach of Templeton (Tree Scanning) to evaluate association between individual SNPs, all SNPs at a gene, and diplotypes, with log-transformed gene expression. SNPs and diplotypes at eight candidate genes exhibited statistically significant (p < 0.05) association with gene expression. Using the literature as a "gold standard" to compare 14 genes with data from both this study and the literature, we observed 80% and 85% concordance for genes exhibiting and not exhibiting significant *cis *sequence effects in our study, respectively.

**Conclusion:**

Based on analysis of our results and the extant literature, one in four genes exhibits significant *cis *sequence effects, and for these genes, about 30% of gene expression variation is accounted for by *cis *sequence variation. Despite diverse experimental approaches, the presence or absence of significant *cis *sequence effects is largely supported by previously published studies.

## Background

Among heritable factors that influence phenotypic expression are sequence polymorphisms in genic regions that affect gene expression rather than protein structure [1,2]. The influence of sequence variation linked to the gene sequence on the regulation of gene expression (*cis *sequence effects) has been studied experimentally in *H. sapiens *at single genes for decades [3], and, more recently, in various multi-gene approaches in *S. cerevisiae *[4-6], *S. purpuratus *[7,8], *D. melanogaster *and *D. simulans *[9,10], *M. musculus *[11,12], *Z. mays *[12], and *H. sapiens *[12-24]. In studies with human tissues, these efforts have characterized *cis *sequence effects on gene expression as common and heritable [13] and have used both unrelated and related individuals to quantify such *cis *sequence effects [15,18]. Array-based genotyping and gene expression platforms [24-27] have been essential for multi-gene approaches, and to generate data enabling investigation of the potential effect of sequence variation not linked to the gene on gene expression (*trans *sequence effects).

We used previously generated genomic resequencing data and, for this study, quantified *in vitro *transcript levels from thirty unrelated individuals at several hundred candidate genes commonly studied in cancer research. We identified a subset of candidate genes with abundant sequence and gene expression variation. We evaluated potential *cis *sequence effects using individual single nucleotide polymorphisms (SNPs), all SNPs at a candidate gene considered jointly and haplotype phylogenies and diplotypes. We compared our findings to the published *cis *sequence effects literature and to the existing gene expression regulation literature available for those candidate genes that exhibited *cis *sequence effects.

## Results

### Gene expression data quality

Thirty lymphoblastoid cell lines drawn from the SNP500Cancer resource were cultured in triplicate and total RNA extracted [see Additional file 1]. Gene expression profiling was performed on the N = 90 samples using a custom Illumina Sentrix^® ^Array Matrix-96 microarrays containing 50 mer probes targeting 697 genes relevant to cancer research [see Additional file 2]. Gene expression data from one array were excluded from further analysis due to a within individual cell line linear r^2 ^correlation for all genes of <95%, while all remaining within individual cell line correlations were ~99%. This correlation statistic reflects variation at all levels of the experiment: cell culture, RNA extraction, RNA labeling, and array performance. Based on these high quality data, further analysis used normalized mean gene expression signal data from the three replicate arrays.

### Selection of genes for analysis of potential cis effects

We used two threshold criteria to select genes from the N = 697 genes for further analysis: a signal amplitude of ≥100 normalized units (38% of the genes); and a between cell line coefficient of variation (CV_IC_) of ≥20% (32% of the genes). These criteria identified N = 95 genes with sufficient, and sufficiently variable, gene expression for further analysis. We used the criterion of ≥2 SNPs per candidate gene in the N = 30 DNAs to identify N = 522 candidate genes with sufficient sequence variation for analysis. We then compared the N = 95 genes with sufficient gene expression variance and N = 522 genes with sequence variation derived from the SNP500Cancer resequencing program, a component of the Cancer Genome Anatomy Project of the National Cancer Institute [28,29] and identified a subset of N = 32 genes (4.6% of the total sample) for the analysis of potential *cis *sequence effects (Table 1).

**Table 1:** Genes chosen for analysis of *cis *sequence effects on gene expression

**Gene**	**Mean Normalized Signal**	**Stand Dev**	**Intra line CC (%)**	**Inter line CV (%)**	**Size (bp)**	**N SNPs available**
ALDH2	134.3	84.2	94.9	63.6	43,438	4
BCL2L1	274.8	79.1	62.8	26.2	58,393	3
BIC	2451.0	585.9	63.9	22.2	12,968	22
BIRC3	813.4	244.4	70.1	27.6	20,271	2
BLM	177.3	59.4	81.4	32.7	97,996	24
CCNA2	343.7	111.1	66.1	27.3	6,367	4
CCND3	807.7	257.4	86.3	30.5	6,880	6
CDKN1B	161.2	44.6	75.4	24.0	4,994	3
CHEK1	173.2	62.4	75.9	31.2	29,300	2
CYP1B1	310.7	206.2	90.6	66.2	8,546	50
EGR1	145.9	73.2	71.4	45.9	3,823	2
FCGR2B	149.2	56.6	85.0	36.9	14,867	9
GADD45A	314.5	90.2	82.5	27.9	3,136	4
IFNGR1	662.0	272.4	92.1	40.4	21,885	2
IFNGR2	852.5	224.8	85.2	24.3	34,624	5
IRF1	267.4	57.3	64.7	20.3	7,647	5
JAK1	161.5	44.2	71.7	25.1	51,778	20
LMO2	187.4	124.5	97.1	68.4	33,711	8
LTA	2196.5	667.3	83.5	29.8	2,005	7
MGMT	417.0	131.5	93.8	31.3	230,861	6
MSH2	190.0	52.5	66.8	23.6	80,097	3
MYBL2	277.6	116.7	74.8	36.4	49,413	25
MYC	512.4	191.1	85.9	34.9	5,170	2
NBS1	163.8	40.4	70.4	23.6	51,187	19
OAS1	490.8	153.5	86.3	30.2	12,956	5
PCNA	2523.3	685.6	71.0	23.6	11,669	3
PHB	178.4	44.3	64.9	20.9	10,822	3
PIM1	145.6	51.2	74.3	33.0	5,218	7
PTEN	297.7	65.5	71.8	20.1	103,207	5
TNF	1097.3	246.4	83.4	22.2	2,762	12
TP73L	110.1	89.0	97.0	81.0	265,849	26
TYMS	1554.5	399.1	69.8	22.9	15,841	19

### Gene expression of selected genes

The mean (standard deviation) rank-invariant normalized gene expression signal of the subset of N = 32 genes was 580 (175) units (Table 1), the mean (standard deviation) intra cell line replicate culture correlation coefficient was 0.78 (0.11), and the mean (standard deviation) between cell line coefficient of variation (CV_IC_) was 0.33 (0.15). Mean noise (3 standard deviations of 20 negative control probes built into the array) was 20 units +/- 4 units. PCNA had the largest mean gene expression, TP73L had the smallest gene expression and the largest CV, and PTEN had the smallest CV.

### Selection of SNPs at genes for association analysis

The number (mean, standard deviation) of polymorphic SNPs per gene ranged from 2 to 50 (9.9, 10.6) and the number of tag (minor allele frequency minimum of >5% and with an r^2 ^threshold ≥ 0.80) and singleton SNPs per gene ranged from 0 to 15 (4.3, 3.7) in the group of N = 32 genes selected for association analysis (Table 2). Two genes (EGR1 and GADD45A) were excluded from further analysis as there were no SNPs at these genes with minor allele frequencies >5%. Two SNPs with genotype completion (attempted/completed) rates of 63% and 77% were excluded. After these exclusions, there were N = 126 tag and singleton SNPs at 30 genes available for analysis with a mean (standard deviation) genotype completion rate of 98.3% (1.1%) [see Additional file 3]. The distribution of SNP genotypes in the N = 30 cell lines was evaluated for Hardy Weinberg Equilibrium using asymptotic and exact tests; there were no SNPs exhibiting exact test P values < 0.01, and two exhibiting exact test P values < 0.05, both were at TP73L. The distribution of the flanking, untranslated region, coding and intronic SNPs was 27%, 15%, 12% and 46%, respectively. Four genes (IRF1, MSH2, MYC, OAS1) had only one informative tag or singleton SNP and were excluded from haplotype-based analyses, leaving N = 26 genes with a range of 2 to 14 tag and singleton SNPs for haplotype based analyses. The gene with the largest number of tag and singleton SNPs (CYP1B1, N = 14 tag and singleton SNPs) generated too many terms for the implementation of the SAM model to compute. The remaining N = 25 genes, comprised of N = 108 tag and singleton SNPs (range 2–10 SNPs/gene), have results from all SNP and haplotype based methods. The number of SNPs before and after selection of tag and singleton SNPs were uncorrelated (Spearman's rho) with mean normalized signal, the standard deviation, intra cell line correlation coefficient, or inter cell line coefficient (data not shown).

**Table 2:** SNPs & haplotypes chosen for analysis of *cis *sequence effects

**Gene**	**N SNPs in N = 30 Cauc sample**	**N tag or singleton SNPs**	**N tag SNPs**	**N singleton SNPs**	**N haplotypes in trees**
ALDH2	4	2	1	1	3
BCL2L1	3	2	0	2	3
BIC	22	9	3	6	8
BIRC3	2	2	0	2	3
BLM	24	9	5	4	7
CCNA2	4	3	0	3	4
CCND3	6	4	0	4	7
CDKN1B	3	3	0	3	6
CHEK1	2	2	0	2	3
CYP1B1	50	14	7	7	19
EGR1	2	0	0	0	na
FCGR2B	9	6	1	5	10
GADD45A	4	0	0	0	na
IFNGR1	2	2	0	2	3
IFNGR2	5	2	0	2	3
IRF1	5	1	1	0	na
JAK1	20	5	2	2	7
LMO2	8	8	0	8	13
LTA	7	10	4	6	9
MGMT	6	4	2	2	6
MSH2	3	1*	na	na	na
MYBL2	25	8	4	4	6
MYC	2	1*	na	na	na
NBS1	19	4	2	2	7
OAS1	5	1	1	0	na
PCNA	3	2	0	2	3
PHB	3	3	0	3	4
PIM1	7	2	1	1	3
PTEN	5	3	0	3	4
TNF	12	10	4	6	9
TP73L	26	9	3	7	13
TYMS	19	4	3	1	4

*There was one SNP with maf > 5% at these two genes; tag SNP analysis was not performed. The single SNP with maf > 5% at each gene was used for SNP-based analyses.

### Significant *cis *sequence effects identified via three association methods

**Figure 1:**
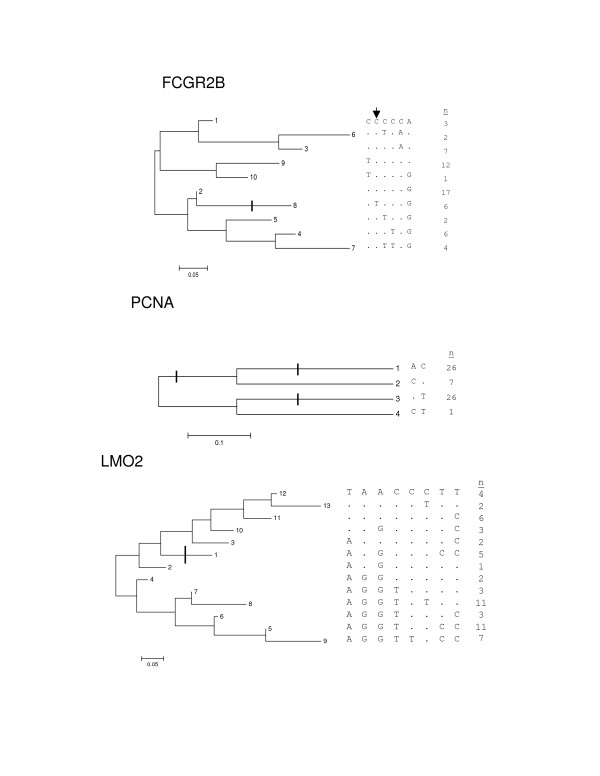
**Haplotype phylogenies and significant haplotype partitions at FCGR2B, PCNA and LMO2**. Haplotype phylogenies are represented together with the SNP allele configuration and the count of haplotypes in the sample. Statistically significant haplotype partitions in the phylogeny are indicated by a vertical or horizontal bar, while an arrow indicates a SNP that exhibits significant association via regression analysis. The haplotypes at FCGR2B, LMO2 and PCNA were constructed using the following SNPs: rs12145988, rs17412751, rs922087, rs2298020, rs1674761, rs844; rs17352 and rs25406; and rs3740616, rs3740617, rs2273797, rs2038602, rs9282776, rs3781577, rs3758640, rs3758641.

N = 10 individual SNPs at six genes were significantly associated with gene expression signal, three genes exhibited significant association to gene expression in the single additive model, and three genes exhibited one or more statistically significant haplotype partitions (Table 3, Figure 1, Additional file 3). The distribution of flanking, untranslated region, coding and intronic SNPs significantly associated to gene expression (30%, 10%, 10% and 50%, respectively) was not significantly different from the distribution of SNPs not exhibiting association to gene expression (Fisher exact test, data not shown). Three (BIC, FCGR2B and NBS1) of the six genes identified using individual SNPs were identified by one or both of the two gene-based association methods. Three genes (BIC, MYBL2, and PCNA) exhibit significant association in one or two methods and only a trend towards statistical significance in another method.

**Table 3:** SNP and haplotype-based *cis *sequence effects on gene expression*

**Gene**	**N SNPs analyzed SNP-wise**	**SAM model analysis, single P**	SNP-wise regression, best P**	**TreeScan, best P_permuted_**
ALDH2	2	0.482	0.577	0.796
BCL2L1	2	0.283	0.488	0.191
BIC	9	**0.010**	**0.016**	0.056
BIRC3	2	0.110	0.550	0.092
BLM	9	0.941	0.266	0.882
CCNA2	3	0.142	0.085	0.635
CCND3	4	0.601	0.519	0.819
CDKN1B	3	0.142	0.059	0.259
CHEK1	2	0.542	0.290	0.577
CYP1B1	14	did not compute	**0.032**	0.438
FCGR2B	6	**0.011**	**0.003**	**0.024**
IFNGR1	2	0.981	0.854	0.902
IFNGR2	2	0.175	0.096	0.374
IRF1	1	0.160	0.282	Na
JAK1	5	0.194	0.375	0.123
LMO2	8	0.830	0.181	**0.024**
LTA	10^3^	0.785	0.380	0.992
MGMT	4	0.330	0.064	0.329
MSH2	1	0.388	0.409	Na
MYBL2	8	0.105	**0.008**	0.095
MYC	1	0.303	0.409	Na
NBS1	4	**0.005**	**0.000**	0.197
OAS1	1	0.186	0.206	Na
PCNA	2	0.138	0.064	**0.006**
PHB	3	0.902	0.651	0.937
PIM1	2	0.689	0.419	0.721
PTEN	3	0.257	0.236	0.569
TNF	10^3^	0.619	0.394	0.756
TP73L	9	0.305	**0.024**	0.563
TYMS	4	0.127	0.100	0.185

*Statistically significant results are bolded. **See Additional file 3 for SNP details.

### SNPs in hybridization probes

We evaluated whether the 50 base pair oligonucleotide probe sequences for the eight genes that exhibited significant *cis *sequence effects exhibit sequence variation in the N = 30 individuals in this study [see Additional file 1]. There were two probes on the array for each gene, and all but two of the sequences (MYBL2 probe IDs = 2099, 3155) have been sequenced in the SNP500Cancer DNAs. Two of the sixteen probe sequences exhibit variation in dbSNP (probe ID = 2168 at CYP1B1 and probe ID = 3562 at PCNA), however neither exhibits variation in the N = 30 DNA samples investigated here.

### Literature data on *cis *sequence effects at candidate genes in this study

We reviewed the multi-gene *cis *sequence effects literature that has investigated either allelic imbalance within individuals (AI), generally defined as expression ratios ≥1.5 or ≤0.67 at transcribed heterozygous SNPs, or SNP-wise linkage or association in related or unrelated samples of individuals, to gene expression [12-24]. We compared results at the N = 14 genes studied in common by this study and at least one additional multi-gene *cis *sequence effects study [18,20-22,24] (Supplementary File 4). To avoid issues of publication bias, we did not compare our results to the single gene *cis *sequence effects literature.

## Discussion

### The proportion of genes exhibiting *cis *sequence effects

We identified nineteen publications that have investigated *cis *sequence effects in a multi-gene approach, one of which summarizes results from six other publications [12-24]. These thirteen publications report results at an average of 457 genes (median of 247, range of 13 to 2,726 genes, standard deviation of 724). The weighted average proportion of genes tested in these thirteen multi-gene studies considered by their authors to exhibit allelic imbalance or statistically significant *cis *sequence effects is 26.2% (unweighted average is 25.7%). We observe statistically significant *cis *sequence effects at eight of thirty genes (26.7%) in our study, which is similar to that observed in the literature.

### The proportion of gene expression attributable to *cis *sequence effects

The literature presents a variety of linkage and association techniques to estimate the proportion of gene expression variation accounted for by individual SNPs [15,17,18,22], expressed either as an allelic ratio [15,18] or as a proportion of variance explained [17,22]. Mean r^2 ^estimates from N = 14 [17] and N = 62 [22] genes exhibiting significant *cis *sequence effects are 35% and 27%, respectively. The average proportion (standard deviation) of gene expression variance explained by individually significant SNPs in the SNP-wise regression analysis [see Additional file 3] in this study was 21% (7%). The average proportion of gene expression variance (standard deviation) explained by the most significant haplotype partitions at FCGR2B, LMO2 and PCNA was 26% (7%). Thus, current technical approaches suggest that approximately one-quarter to one-third of gene expression variation is attributable to *cis *sequence effects.

### Concordance of the multi-gene *cis *sequence effects literature and this study

Pant et al., 2006 [Pant et al., 2006] examined eight genes for *cis *sequence effects in common with our study, the largest overlap in the extant literature [see Additional file 4]. Both studies observed significant *cis *sequence effects at CYP1B1, FCGR2B and MYBL2. Pant et al., observed AI at TYMS where we did not, while we observed *cis *sequence effects at LMO2 and NBS1, where Pant et al. did not. Neither study observed *cis *sequence effects at JAK1 and MYC. In toto, among the 14 genes jointly analyzed in this study and in the literature, and after excluding two genes with discordant literature results, 4 of 5 genes concordantly exhibit, and 6 of 7 genes concordantly do not exhibit, significant *cis *sequence effects, respectively. Concordance with results obtained using other experimental methods, as we have observed here, increases confidence in our results. Such evidence may provide the justification to proceed to more focused functional investigation of gene regulation.

### The utility of different statistical methods used to evaluate *cis *sequence effects

We used three methods to test for the significance of *cis *sequence effects, after restricting the number of SNPs to tag and singleton SNPs at each gene. Regression of individual SNPs identified the largest number of genes exhibiting significant *cis *sequence effects and is the most commonly used method in the literature, after assessment of AI. Mander's single additive method (SAM) [30,31] and Templeton's Tree Scanning method [32,33] identified three genes; one gene, FCGR2B, was identified in common. The latter two methods incorporate correction for multiple tests in their estimates of the significance of association. Rather than apply any formal correction(s) for the multiple comparisons at a gene after regression analysis on SNPs, or after using multiple methods, we compare and contrast the results obtained from using the three methods. Differences between results obtained analyzing individual SNPs and the two methods that apply multiple test correction at the level of the gene suggests that much of the evidence for significant *cis *sequence effects in this sample of N = 30 LCLs is too weak to survive multiple test correction, emphasizing the necessity to apply multiple test corrections to avoid elevated Type I error [22]. We also observed two examples of a gene exhibiting significant *cis *sequence effects with Tree Scanning, but not with the additive methods. At LMO2, the minor alleles of four SNPs define a haplotype partition that exhibits significantly reduced gene expression, and at PCNA, the heterozygote diplotype exhibits significantly increased gene expression [see Additional file 4]. These latter findings depend upon the ability to model the effects of haplotypes within and between individuals. We observed dominant effects for haplotypes at FCGR2B and LMO2 and heterotic effects for haplotypes at PCNA, suggesting that searching for *cis *sequence effects using only additive model-based approaches may result in elevated Type II error rates.

### Biological relevance of *cis *sequence effects – FCGR2B as an example

The focus of this study is to evaluate association between variation in DNA sequence and *in vitro *RNA transcription in a group of candidate genes commonly studied in cancer research. We briefly review some of the recent functional genomics literature for the candidate gene FCGR2B [see Additional file 5] and suggest below how a review of relevant genomic data and our *cis *sequence effects findings at FCGR2B might inform our understanding of this literature, as an example of how our findings might influence future FCGR2B SNP association or functional analyses. The SNP500Cancer program resequenced portions of IVS1, Exon 2, IVS2, IVS6 and Exon 8 of FCGR2B, yielding N = 9 FCGR2B SNPs available with a minor allele frequency of ≥5% for analysis of gene expression variation. After selecting one tag and five singleton SNPs to reduce the number of statistical tests performed with minimal loss of information [34], significant cis sequence effects were observed at FCGR2B SNP rs17412751 (IVS1-91C>T) (Table 3 and Additional file 4). The minor allele frequency of rs17412751 in our sample of N = 30 was 10%, similar to the minor allele frequencies of FCGR2B promoter and transmembrane SNPs previously studied, however, it should be noted that rs17412751 is a singleton SNP, i.e., not strongly associated with the other SNPs available at FCGR2B. Linkage disequilibrium (LD) within the FCGR2B genomic region in Caucasian samples extends from the 3' end of IVS 1 distally in a punctate fashion, and there is some evidence for a separate 5' region of LD proximal to Exon 1 (data not shown). rs17412751 has been genotyped by Hinds et al., 2005 as afd1120510 [35] with a minor allele frequency of 11%, and within their sample of N = 24 European American DNAs, this SNP exhibits strong LD with one additional SNP (rs17404379, afd1120529). However, both of these SNPs map to both intronic regions within the FCGR2B locus and also within the FCGR3B/FCGR3A intergenic region some 70 kbp proximal, suggesting that high sequence homology may be interfering with accurate map assignment. There are two recent reports of copy number variation (CNV) in the region that are relevant: CNV of the FCGR3B locus is associated with nephritis in a rat model and in human patients [36], and analysis of SNP genotypes and genomic hybridization with the HapMap sample has identified a 256 Kbp region as human copy number locus CNV_ID_62 containing the FCGR2A, HSPA6, FCGR3A, FCGR2B and FCRLM1 loci [37].

Our data at FCGR2B is concordant with data generated from both *in vitro *and *ex vivo *experimental strategies that sequence variation in the promoter [38,39] and the coding region [24] is associated with gene expression differences. Further, our data contributes to the evidence that a minor allele frequency of ~10% characterizes the SNPs that are associated with FCGR2B gene expression differences. The inconsistent directionality of effect of the minor allele may be due to high sequence homology at the CNV_ID_62 locus affecting the physical and linkage disequilibrium mapping of the region, or may be due to incomplete linkage disequilibrium between the analyzed SNP and a unanalyzed SNP that may be causing the effect. Individuals wishing to investigate regulation of gene expression at FCGR2B in the future should include approaches necessary to characterize the physical, linkage disequilibrium, transcriptional and copy number architecture of the region.

### Strengths and limitations

Strengths of this study include: high quality gene expression data from triplicate cell cultures for each lymphoblastoid cell line (LCL), with standardization of culture, RNA extraction, labeling, amplification and hybridization conditions; the use of sequence-verified SNPs and the resequencing of nearly all expression array probe sequence regions; the use of multiple methods to evaluate evidence for significant *cis *sequence effects; and comparison of the results observed in this study to the published *cis *sequence effects literature at both the gene and SNP levels.

Limitations of this study include: the use of Epstein-Barr virus-transformed LCLs; the modest number of LCLs used; the association paradigm; and the absence of genetic assays that evaluate copy number in our sample. Limitations of LCLs as reagents for the investigation of gene expression regulation include gene expression in a virally transformed surrogate *ex vivo *tissue, which may influence and potentially eliminate Epstein-Barr virus infection associated genes from gene expression investigation [40,41]. Systematic comparison of gene expression from LCLs and non-surrogate, minimally processed tissues, e.g., peripheral blood lymphocytes, could be an approach towards validation of gene expression findings made in LCLs [40]. The modest number of cell lines used in this study limits statistical power, and is less than the number of cell lines used by some researchers [20-22], however, the number of genes evaluated was also modest. The association paradigm suffers from well known limitations [42]. Some publications testing large number of genes for *cis *sequence effects do not provide complete lists of genes tested or of genes exhibiting significant *cis *sequence effects on gene expression, therefore, we could not identify all genes studied in these reports. Also, due to the variety of approaches used in the literature, most comparisons are between categorical results of specific assays, i.e., it is generally not possible to compare quantitative data from different studies. The MYBL2 probe sequence regions were not resequenced in the N = 30 Caucasian DNA samples and thus the positive regression and TreeScan results at MYBL2 could potentially be a false positive result due to an unidentified SNP within the sequence complementary to these probes.

## Conclusion

We tested for significant association between gene sequence variation and gene expression variation at N = 30 candidate genes in DNA and RNA from N = 30 LCLs. Significant association between *cis *sequence and gene expression variation was observed in 8 out of 30 genes, and accounted for 26% of gene expression variation in three genes evaluated using an analysis of variance approach. We utilized additive and analysis of variance (guided by haplotype phylogeny) analytical approaches, and suggest that approaches that permit modeling of allelic effects may identify effects missed by additive models, although larger multi-gene studies would clarify the relative utility of the two approaches. We reviewed the multi-gene *cis *sequence effects literature and found data on N = 14 of the candidate genes that we evaluated; most of that data is concordant with our results. Investigators using current technologies should expect to find *cis *sequence effects at about one quarter of candidate genes evaluated: these effects will explain about one quarter of gene expression variance. SNPs associated with gene expression can be preferentially selected for genotyping and analysis in genetic association studies, or nominated for functional genomic investigations to characterize their role in the regulation of gene expression.

## Methods

### Preparation of total RNA

For this study, we cultured N = 30 Coriell Cell Repository LCLs [see Additional file 1], in triplicate under standardized conditions; when cells per milliliter exceeded 2 × 10^7^, the cells were harvested, the pellets were washed once with PBS and frozen at -80°C. Same-lot cell culture reagents were used, with three technicians each dedicated to culturing N = 10 of these cell lines, with replicate cell cultures cultured in series. Total RNA was prepared from frozen cell pellets using the Qiagen RNeasy Midi-kit (Valencia, CA). Ten cell culture pellets were extracted at a time by a single technician with a single assistant; cell pellets were removed one-at-a-time from the -80°C freezer, quick thawed by rubbing between gloved hands, and Qiagen denaturant immediately added. Ethanol was added to each sample, vortexed, and the samples applied to Qiagen Midi columns, washed as specified, treated with RNase-free DNase "on-column", followed by additional washes before elution of the RNA with the provided buffer. After elution, sample volume was determined by weight, sodium acetate was added to 0.3 M, the sample was split and ethanol added at 3× volume to each aliquot and stored at -20°C.

### Gene expression

We performed gene expression experiments RNA from the N = 90 cell cultures using custom Illumina Sentrix^® ^Array Matrix-96 microarrays containing 50 mer probes targeting 697 genes relevant to cancer research [see Additional file 2]. Candidate gene content for the custom array was developed by a voting scheme based on other "cancer gene" lists available on the World Wide Web on November 6, 2003. Eleven lists were assembled from a variety of academic, industrial and government sources and a list of all genes present on all lists was assembled with the genes ranked according to frequency of occurrence. Any gene appearing ≥ four lists was included in the array design [see Additional file 2]. We designed the custom microarray using transcript sequences from NCBI RefSeq build 34.3. Sequences overlapping SNPs as defined in dbSNP build 121 and SNP500Cancer were avoided. RNA samples were amplified and labeled by the method of Eberwine [43], using the MessageAmp aRNA kit (Ambion, Inc., Austin, TX). Specific conditions of labeling, as well as array hybridization, washing and staining, and data extraction and processing were performed as described in Kuhn et al., 2004 [26], as were array processing and data extraction and processing. Array hybridization intensity signals were adjusted using a global background subtraction and rank-invariant normalization algorithm. All gene expression data generated for this experiment has been deposited in the Gene Expression Omnibus with Series accession ID = GSE8394.

### SNP, haplotypes and haplotype phylogeny

We utilized sequence verified SNP data from N = 30 individuals from the Caucasian subsample of the SNP500Cancer database [28,29], which currently contains sequence data from >750 genes that have been partially resequenced in a sample of N = 102 DNAs. We selected SNPs as either tag SNPs or singleton SNPs using a minor allele frequency minimum of >5% and with an r^2 ^threshold ≥ 0.80. We reconstructed haplotypes using PHASE [44] using the tag and singleton SNPs with the following parameters: number of iterations = 10,000; thinning interval = 1; burn-in = 10,000. We performed haplotype phylogeny reconstruction using neighbor-joining with a uniform model of genetic distance in MEGA version 3.1 [45]. We searched for SNPs in genomic sequence complementary to probe sequence using Genewindow [46], using data from NCBI genome build 35.1, dbSNP build 125, and the SNP500Cancer resequencing program.

### Association analysis with gene expression

We managed gene expression and SNP and haplotype data and performed descriptive analysis in STATA 9.0 (StataCorp LP, College Station, TX). We evaluated normality of gene expression using the Shapiro-Wilk test, log-transformed gene expression and used log-transformed values in each analysis method. The coefficient of variation between cell lines (CV_IC_) was calculated as follows CV_IC _= (SD_IC_/μ)*100, where SD_IC _is the standard deviation between cell lines estimated from a one-way analysis-of-variance model, and μ is the mean expression of the gene. The first analysis used linear regression, modeling gene expression as a function of each SNP separately, using an additive model to test for a trend across genotypes. The second analysis included all SNPs in a gene simultaneously and compared that model to a model without any predictors by means of a likelihood ratio test [30,31]. Third, we partitioned a haplotype phylogeny of each candidate gene to construct partition diplotypes and performed one-way ANOVA analyses of the quantitative gene expression trait associated with these partition genotypes to search for partitions that explain a statistically significant proportion of gene expression variation [32], using the software TreeScan [33]. We report the proportion of the gene expression variance explained by the partition diplotypes and the P value from the F statistic after correction by permutation and enforcement of monotonicity. While TreeScan performs a second round of testing for significant partitions conditional on partitions identified in the first round of analysis, no additional significant partitions were identified upon conditional analysis in this dataset. In this study, all P values are two-sided and must be <0.05 to be considered significant.

## Abbreviations

AI: Allelic Imbalance. ANOVA: Analysis of Variance. CEPH: Centre d'Etude du Polymorphisme Humain. CV: Coefficient of Variation (CV). DNA: Deoxyribonucleic Acid. LCL: Lymphoblastoid cell line. Mbp: Megabasepair. SAM: Single Additive Model. SNP: Single Nucleotide Polymorphism. RNA: Ribonucleic Acid.

## Authors' contributions

AWB designed the experiments, nominated genes for potential inclusion in the array, participated in data analysis of the gene expression data, and drafted and revised the manuscript. AB performed data analysis of gene expression data and helped revise the manuscript. TM selected the custom Illumina Sentrix^® ^Array Matrix-96 array gene content and participated in experimental design and data analysis. KK performed the gene expression experiments and participated in experimental design and data analysis. JK designed the array probes. RP provided guidance on all statistical procedures and revised the manuscript. PB participated in experimental design and directed cell culture, RNA extraction and quality control analysis of total RNA. KJ provided analytical support on tag and singleton SNP selection and HWE testing. BP provided downloads of the SNP500Cancer database for probe design of the gene expression array and for selection of genes with sequence variation in the SNP500Cancer Caucasian sample. SC participated in experimental design, nominated genes for potential inclusion in the array, and helped revised the manuscript. MY participated in experimental design, nominated genes for potential inclusion in the array, performed haplotype and phylogenetic reconstruction of SNP500Cancer candidate gene SNP data and helped interpret the results of tree scanning association analysis.

## Supplementary Material

Additional file 1 Illumina probes targeting 697 cancer research candidate genes Click here for file

Additional file 2 N = 30 Coriell Cell Repository cell lines used for analysis Click here for file

Additional file 3 Annotated SNP list at N = 30 candidate genes evaluated for *cis *sequence effects Click here for file

Additional file 4 Comparison of *cis *sequence effects in this study with results from the literature Click here for file

Additional file 5 Genetic epidemiology and functional genomics of FCGR2B SNPs Click here for file
