# Supplementary material for: cis sequence effects on gene expression
Source: BMC Genomics. 2007 Aug 29;8:296. doi: 10.1186/1471-2164-8-296 (PMC2077339; doi:10.1186/1471-2164-8-296)
Supplement: Additional file 2 — N = 30 Coriell Cell Repository cell lines used for analysis [file 1471-2164-8-296-S2.doc]

| **#** | **NCP ID (SNP500Cancer)** | **GM ID (Coriell)** | **NA ID (Coriell)** | **Cell lines ID as provided by Coriell** |
| --- | --- | --- | --- | --- |
| 1 | NCP072 | GM06987 | NA06987 | GM06987D |
| 2 | NCP073 | GM07038 | NA07038 | GM07038D |
| 3 | NCP074 | GM07348 | NA07348 | GM07348D |
| 4 | NCP075 | GM07349 | NA07349 | GM07349A |
| 5 | NCP076 | GM10831 | NA10831 | GM10831 |
| 6 | NCP077 | GM10849 | NA10849 | GM10849 |
| 7 | NCP079 | GM11522 | NA11522 | GM11522 |
| 8 | NCP080 | GM11523 | NA11523 | GM11523 |
| 9 | NCP081 | GM11524 | NA11524 | GM11524 |
| 10 | NCP082 | GM11525 | NA11525 | GM11525 |
| 11 | NCP083 | GM11993 | NA11993 | GM11993 |
| 12 | NCP084 | GM12273 | NA12273 | GM12273 |
| 13 | NCP085 | GM12749 | NA12749 | GM12749 |
| 14 | NCP086 | GM12813 | NA12813 | GM12813 |
| 15 | NCP087 | GM12841 | NA12841 | GM12841 |
| 16 | NCP088 | GM12863 | NA12863 | GM12863 |
| 17 | NCP089 | GM12909 | NA12909 | GM12909 |
| 18 | NCP090 | GM12911 | NA12911 | GM12911 |
| 19 | NCP091 | GM12912 | NA12912 | GM12912 |
| 20 | NCP092 | GM13617 | NA13617 | GM13617 |
| 21 | NCP093 | GM13618 | NA13618 | GM13618 |
| 22 | NCP094 | GM13619 | NA13619 | GM13619 |
| 23 | NCP118 | GM06990 | NA06990 | GM06990D |
| 24 | NCP119 | GM07057 | NA07057 | GM07057D |
| 25 | NCP120 | GM10858 | NA10858 | GM10858 |
| 26 | NCP121 | GM10859 | NA10859 | GM10859 |
| 27 | NCP122 | GM10860 | NA10860 | GM10860 |
| 28 | NCP123 | GM10861 | NA10861 | GM10861 |
| 29 | NCP124 | GM10832 | NA10832 | GM10832 |
| 30 | NCP125 | GM10833 | NA10833 | GM10833 |
