# Supplementary material for: cis sequence effects on gene expression
Source: BMC Genomics. 2007 Aug 29;8:296. doi: 10.1186/1471-2164-8-296 (PMC2077339; doi:10.1186/1471-2164-8-296)
Supplement: Additional file 4 — Comparison of cis sequence effects in this study with results from the literature [file 1471-2164-8-296-S4.doc]

**Comparison of *cis* sequence effects in this study with results from the literature**

Pant et al., 2006 [1] examined N=1,983 SNPs from N=1,389 genes using non-transformed lymphocyte RNA, with an average of N=502 heterozygous SNPs at N=418 genes for each of N=12 DNA samples and where 22% of these SNPs (25% of the genes) exhibited AI. They evaluated N=12 SNPs and we evaluated four of the same SNPs and N=46 additional SNPs at eight genes in common (CYP1B1, FCGR2B, JAK1, LMO2, MYBL2, MYC, NBS1 and TYMS). They observed AI at SNPs at CYP1B1, FCGR2B, MYBL2 and TYMS, while we observed statistically significant *cis* sequence effects at CYP1B1, FCGR2B, LMO2, MYBL2 and NBS1. The definition of differential expression (AI) in Pant et al., 2006 is if the allele frequency fold ratio (reference to alternative allele) in heterozygote individuals is ≥1.5 or ≤0.67).

Both CYP1B1 SNPs investigated by Pant et al., 2006 exhibited differential expression expressed by an allele frequency ratio (reference to alternate) of ~50%. One of these two SNPs, rs1056837, was investigated in our study, but did not exhibit statistically significant *cis* sequence effects. One (rs151313) of the fourteen CYP1B1 SNPs that we investigated exhibited statistically significant *cis* sequence effects (P=0.0316).

FCGR2B SNP rs1050501 (Ex5+49, I232T) exhibited AI in Pant et al., 2006, with the reference allele exhibiting ≥10 fold increased fluorescence over the alternate allele. While this SNP was not investigated in our study, one of the six FCGR2B SNPs that we investigated (rs17412751, IVS1-91) exhibited statistically significant *cis* sequence effects (P=0.0033). At FCGR2B, the six individuals with one copy of haplotype 8, defined by the minor allele of rs17412751, exhibited significantly increased gene expression than the remaining individuals (mean of 5.33 versus 4.84 natural log-transformed normalized units, F=10.34, P=0.0033/0.024 before/after permutation). This haplotype partition accounts for 24% of FCGR2B expression variance.

Both studies investigated and observed statistically significant *cis* sequence effects at MYBL2 SNP rs2070235. Pant et al., 2006 observed increased expression of the reference allele and the minor allele in two different individuals, suggesting that MYBL2 may be subject to imprinting. In our study, this SNP exhibits statistically significant association to gene expression (P=0.0078), the minor allele defines a haplotype that is statistically significantly associated to gene expression prior to significance correction via permutation (P=0.023), which haplotype partition accounts for 14% of MYBL2 expression variance.

The TYMS SNP exhibiting AI in Pant et al., 2006 (rs699517) is 70bp distal and in strong LD (D’=1.0, LOD=9.8 and r^2^=0.60) in the SNP500Cancer Caucasian sample with one of the TYMS SNP that we investigated (rs2790), which SNP exhibits a trend (P=0.0998) towards statistically significant *cis* sequence effects in our study.

Four additional genes (JAK1, LMO2, MYC and NBS1) were tested in both studies; in Pant et al., 2006, none of the seven SNPs evaluated at these genes exhibited AI. In our study, one of the four NBS1 SNPs that we investigated (rs2735383) exhibited statistically significant *cis* sequence effects. At LMO2, the haplotype of interest is defined by the minor alleles of four SNPs (rs3740616, rs2273797, rs3758640, rs3758641), but none of these SNPs exhibit statistically significant or trending SNP-wise association to gene expression. The five individuals with one copy of haplotype 1 exhibited statistically significantly decreased gene expression than the remaining individuals (4.24 vs. 5.15 log-transformed units, P=0.024). This haplotype partition accounts for 21% of LM02 expression variance). The other N=16 SNPs that we examined at these four genes did not exhibit statistically significant *cis* sequence effects, including two SNPs at JAK1 and LMO2 (rs11208529 and rs3740617) that were examined in both studies.

Lo et al., 2003 [2] examined AI in human fetal kidney and liver RNA from seven individuals at N=1,063 transcribed SNPs at N=602 genes using a SNP variant detection array [3]; OAS1 was the single gene that overlapped between their study and our study: they examined rs2660, while we examined rs10774671, 250 base pairs proximal and in allelic identity with rs2660 in the HapMap Caucasian samples – neither SNP exhibited statistically significant *cis* sequence effects.

Pastinen et al., 2004 [4] examined AI at N=193 SNPs in N=129 genes from 63 lymphoblastoid cell lines (LCLs) using HG-U133A Genechips (Affymetrix, Santa Clara, CA), and fluorescence polarization based single-base-extension genotyping and allele ratio estimation [5]. Their sample of SNPs and genes overlapped with three SNPs and five genes from those we studied. They (we) examined the following SNPs at the five genes: IFNGR1, rs11914 (rs11914 and rs3799488); IFNGR2, rs1059293 (rs1059293 and rs9808753); IRF1, rs839 (rs9282761); LTA, rs1041981 (we evaluated 10 different SNPs at the combined LTA/TNF locus); PHB, rs6917 (rs6917, rs1049620 and rs4987082). Neither they nor we observed statistically significant *cis* sequence effects at these SNPs, and we did not observe statistically significant *cis* sequence effects at these five genes.

Pastinen et al., 2005 [6] examined N=121 SNPs at N=64 genes from N=60 Caucasian LCLs from the CEPH HapMap panel with *a priori* evidence for AI for *cis* sequence effects via resequencing, linkage mapping and RT-PCR, and report statistically significant AI for N=18 genes. The only candidate gene that overlapped between their study and our study was OAS1. They observed AI at rs2285934 at OAS1, 5,773 base pairs 5’ and in allelic identity to the OAS1 SNP (rs10774671) that we examined. We did not observe statistically significant *cis* sequence effects at rs10774671.

Stranger et al., 2005 [7] examined N=374 genes from the N=60 Caucasian LCLs from the CEPH HapMap panel for *cis* sequence effects using the same microarray technology that we used to measure gene expression [8], and used N=753,712 HapMap SNPs ([www.hapmap.org](http://www.hapmap.org/)) with the same regression approach that we used to search for statistically significant *cis* and *trans* sequence effects , where testing for *cis* sequence effects was confined to SNPs within a footprint of 1 Mbp surrounding each gene. Based on the SNP density of the HapMap version used (one per 5 kilobasepairs), approximately 200 SNPs may have been used at each gene to evaluate *cis* sequence effects. IFNGR2 was the only gene in a list of N=64 genes reported to exhibit statistically significant *cis* sequence effects that overlapped with our study. We tested two SNPs at IFNGR2 in our study; rs1059293 exhibited a trend towards a statistically significant *cis* sequence effect (P=0.0963).

**Table 1. Comparing cis sequence effects in this study with the multi-gene literature**

| Gene | This study^1^ | Literature multi-gene *cis* sequence effects studies^2^ | | | | |
| --- | --- | --- | --- | --- | --- | --- |
|  |  | [1] | [2] | [4] | [6] | [7] |
| ALDH2 | 0 | 0 | 0 | 0 | 0 | 0 |
| BCL2L1 | 0 | 0 | 0 | 0 | 0 | 0 |
| BIC | 1 | 0 | 0 | 0 | 0 | 0 |
| BIRC3 | 0 | 0 | 0 | 0 | 0 | 0 |
| BLM | 0 | 0 | 0 | 0 | 0 | 0 |
| CCNA2 | 0 | 0 | 0 | 0 | 0 | 0 |
| CCND3 | 0 | 0 | 0 | 0 | 0 | 0 |
| CDKN1B | 0 | 0 | 0 | 0 | 0 | 0 |
| CHEK1 | 0 | 0 | 0 | 0 | 0 | 0 |
| CYP1B1 | 1 | 2 | 0 | 0 | 0 | 0 |
| FCGR2B | 1 | 2 | 0 | 0 | 0 | 0 |
| IFNGR1 | 0 | 0 | 0 | 1 | 0 | 0 |
| IFNGR2 | 0 | 0 | 0 | 1 | 0 | 2 |
| IRF1 | 0 | 0 | 0 | 1 | 0 | 0 |
| JAK1 | 0 | 1 | 0 | 0 | 0 | 0 |
| LMO2 | 1 | 1 | 0 | 0 | 0 | 0 |
| LTA | 0 | 0 | 0 | 1 | 0 | 0 |
| MGMT | 0 | 0 | 0 | 0 | 0 | 0 |
| MSH2 | 0 | 0 | 0 | 0 | 0 | 0 |
| MYBL2 | 1 | 3 | 0 | 0 | 0 | 0 |
| MYC | 0 | 1 | 0 | 0 | 0 | 0 |
| NBS1 | 1 | 1 | 0 | 0 | 0 | 0 |
| OAS1 | 0 | 0 | 1 | 0 | 2 | 0 |
| PCNA | 1 | 0 | 0 | 0 | 0 | 0 |
| PHB | 0 | 0 | 0 | 1 | 0 | 0 |
| PIM1 | 0 | 0 | 0 | 0 | 0 | 0 |
| PTEN | 0 | 0 | 0 | 0 | 0 | 0 |
| TNF | 0 | 0 | 0 | 0 | 0 | 0 |
| TP73L | 1 | 0 | 0 | 0 | 0 | 0 |
| TYMS | 0 | 2 | 0 | 0 | 0 | 0 |

^1^0=Candidate gene examined but did not exhibit significant *cis* sequence effects; 1=Candidate gene examined and exhibits significant *cis* sequence effects.

^2^0=Candidate gene not examined for *cis* sequence effects; 1=Candidate gene tested and does not exhibit significant *cis* sequence effects; 2=Candidate gene tested and exhibits gene-wise *cis* sequence effects but not at the same SNP or SNPs; 3=Candidate gene tested and exhibits *cis* sequence effects with the same SNP or SNPs.

Reference List

1. Pant PV, Tao H, Beilharz EJ, Ballinger DG, Cox DR, Frazer KA: **Analysis of allelic differential expression in human white blood cells.** *Genome Res* 2006, **16:** 331-339.

2. Lo HS, Wang Z, Hu Y, Yang HH, Gere S, Buetow KH *et al*.: **Allelic variation in gene expression is common in the human genome.** *Genome Res* 2003, **13:** 1855-1862.

3. Wang DG, Fan JB, Siao CJ, Berno A, Young P, Sapolsky R *et al*.: **Large-scale identification, mapping, and genotyping of single-nucleotide polymorphisms in the human genome.** *Science* 1998, **280:** 1077-1082.

4. Pastinen T, Sladek R, Gurd S, Sammak A, Ge B, Lepage P *et al*.: **A survey of genetic and epigenetic variation affecting human gene expression.** *Physiol Genomics* 2004, **16:** 184-193.

5. Hsu TM, Chen X, Duan S, Miller RD, Kwok PY: **Universal SNP genotyping assay with fluorescence polarization detection.** *Biotechniques* 2001, **31:** 560, 562, 564-560,8, passim.

6. Pastinen T, Ge B, Gurd S, Gaudin T, Dore C, Lemire M *et al*.: **Mapping common regulatory variants to human haplotypes.** *Hum Mol Genet* 2005, **14:** 3963-3971.

7. Stranger BE, Forrest MS, Clark AG, Minichiello MJ, Deutsch S, Lyle R *et al*.: **Genome-wide associations of gene expression variation in humans.** *PLoS Genet* 2005, **1:** e78.

8. Kuhn K, Baker SC, Chudin E, Lieu MH, Oeser S, Bennett H *et al*.: **A novel, high-performance random array platform for quantitative gene expression profiling.** *Genome Res* 2004, **14:** 2347-2356.
