# Supplementary material for: cis sequence effects on gene expression
Source: BMC Genomics. 2007 Aug 29;8:296. doi: 10.1186/1471-2164-8-296 (PMC2077339; doi:10.1186/1471-2164-8-296)
Supplement: Additional file 5 — Genetic epidemiology and functional genomics of FCGR2B SNPs [file 1471-2164-8-296-S5.doc]

### Genetic epidemiology and functional genomics of FCGR2B SNPs

FCGR2B codes for a low affinity IgG Fc gamma receptor (Fc gamma IIb), which mediates inhibitory immune response signaling [1,2], and is one of a family of inhibitory and activatory Fc gamma receptors involved in the immune response. FCGR2B is a positional and biological candidate gene for a number of diseases, including B cell non-Hodgkin’s lymphoma, with increased FCGR2B transcription observed after chromosomal translocations juxtapose FCGR2B with immunoglobulin loci [3,4], and for autoimmune diseases, including systemic lupus erythematosus (SLE), where the Fc gamma receptor region on chromosome 1q23 region has been repeatedly identified in whole genome linkage analyses of SLE [5-8]. FCGR2B polymorphisms have been associated with SLE in case:control studies [9-14], with differences in FCGR2B gene transcription and protein expression [12,13], and with differences in Fc gamma IIb function [15-17]. Epistasis has been demonstrated in mouse models of SLE [1], and in human SLE case:control association studies [18]. Coordinate regulation of FCGR2A and FCFR2B has been identified in analyses of purified neutrophils from groups of healthy volunteers, [2,19]. FCGR2A and FCFR2B mRNA ratios have been shown to be stable over periods of up to one year, are maintained after cytokine stimulation, and are associated with FCGR2B promoter haplotypes [19]. FCGR2B promoter SNPs have been associated with SLE in Caucasian case:control samples, e.g., at rs3219018 (-385G>C) [13], and at a haplotype composed of alleles at rs10799879 (-892C>G), rs3219018 and rs34701572 (-119T>A) [12]. The minor allele (frequency of 12% in controls and 14% in SLE patients), or minor haplotype (frequency of 9% in controls and 14% in SLE patients), was associated to disease (SLE) status in both studies [12,13], and to gene expression *in vitro* and to protein expression in ex vivo peripheral B cells, although the directionality of the allelic effects were discordant between the two studies [12,13]. The minor allele homozygote and minor allele of rs1050501 (Ex5+49T>C, a non-synonymous polymorphism coding for a I232T substitution in a Fc gamma IIb transmembrane domain), have been significantly associated in multiple Asian case:control samples [9-11,14], but not in African-American or Caucasian case:control samples [15]. The threonine (minor) 232 protein allele has demonstrated reduced inhibitory Fc gamma receptor function using both *in vitro* transfection analyses and *ex vivo* macrophages expressing equivalent amounts of the isoleucine (major) or the minor protein allele [16,17]. These differences are proposed to be due to the inability of the minor allele to inhibit activatory Fc gamma receptor (Fc gamma IIa) dependent signaling through exclusion of the minor allele from intracellular lipid rafts [16,17]. A common cellular phenotype evaluated in the analysis of differences between Fc gamma IIb alleles exhibit significant differences in calcium influx after B cell IgG receptor aggregation, where the average increase, corresponding to a reduction in Fc gamma IIb inhibitory function, was about 1.6 fold (range of 1.2 to 2.2) [15-17]. While highly informative with respect to the functionality of these two Fc gamma IIb alleles *in vitro*, these data do not provide information on the extent to which potential functional differences between the two Fc gamma IIb alleles *in vivo* might be due to gene expression differences [20], because these *in vitro* experiments were performed after normalizing expression of Fc gamma IIb alleles via cell culture conditioning and/or flow cytometry.

Reference List

1. Bolland S, Ravetch JV: **Spontaneous autoimmune disease in Fc(gamma)RIIB-deficient mice results from strain-specific epistasis.** *Immunity* 2000, **13:** 277-285.

2. Pricop L, Redecha P, Teillaud JL, Frey J, Fridman WH, Sautes-Fridman C *et al*.: **Differential modulation of stimulatory and inhibitory Fc gamma receptors on human monocytes by Th1 and Th2 cytokines.** *J Immunol* 2001, **166:** 531-537.

3. Callanan MB, Le Baccon P, Mossuz P, Duley S, Bastard C, Hamoudi R *et al*.: **The IgG Fc receptor, FcgammaRIIB, is a target for deregulation by chromosomal translocation in malignant lymphoma.** *Proc Natl Acad Sci U S A* 2000, **97:** 309-314.

4. Chen W, Palanisamy N, Schmidt H, Teruya-Feldstein J, Jhanwar SC, Zelenetz AD *et al*.: **Deregulation of FCGR2B expression by 1q21 rearrangements in follicular lymphomas.** *Oncogene* 2001, **20:** 7686-7693.

5. Moser KL, Neas BR, Salmon JE, Yu H, Gray-McGuire C, Asundi N *et al*.: **Genome scan of human systemic lupus erythematosus: evidence for linkage on chromosome 1q in African-American pedigrees.** *Proc Natl Acad Sci U S A* 1998, **95:** 14869-14874.

6. Cantor RM, Yuan J, Napier S, Kono N, Grossman JM, Hahn BH *et al*.: **Systemic lupus erythematosus genome scan: support for linkage at 1q23, 2q33, 16q12-13, and 17q21-23 and novel evidence at 3p24, 10q23-24, 13q32, and 18q22-23.** *Arthritis Rheum* 2004, **50:** 3203-3210.

7. Shai R, Quismorio FP, Jr., Li L, Kwon OJ, Morrison J, Wallace DJ *et al*.: **Genome-wide screen for systemic lupus erythematosus susceptibility genes in multiplex families.** *Hum Mol Genet* 1999, **8:** 639-644.

8. Tsao BP, Cantor RM, Grossman JM, Kim SK, Strong N, Lau CS *et al*.: **Linkage and interaction of loci on 1q23 and 16q12 may contribute to susceptibility to systemic lupus erythematosus.** *Arthritis Rheum* 2002, **46:** 2928-2936.

9. Kyogoku C, Dijstelbloem HM, Tsuchiya N, Hatta Y, Kato H, Yamaguchi A *et al*.: **Fcgamma receptor gene polymorphisms in Japanese patients with systemic lupus erythematosus: contribution of FCGR2B to genetic susceptibility.** *Arthritis Rheum* 2002, **46:** 1242-1254.

10. Siriboonrit U, Tsuchiya N, Sirikong M, Kyogoku C, Bejrachandra S, Suthipinittharm P *et al*.: **Association of Fcgamma receptor IIb and IIIb polymorphisms with susceptibility to systemic lupus erythematosus in Thais.** *Tissue Antigens* 2003, **61:** 374-383.

11. Chu ZT, Tsuchiya N, Kyogoku C, Ohashi J, Qian YP, Xu SB *et al*.: **Association of Fcgamma receptor IIb polymorphism with susceptibility to systemic lupus erythematosus in Chinese: a common susceptibility gene in the Asian populations.** *Tissue Antigens* 2004, **63:** 21-27.

12. Su K, Wu J, Edberg JC, Li X, Ferguson P, Cooper GS *et al*.: **A promoter haplotype of the immunoreceptor tyrosine-based inhibitory motif-bearing FcgammaRIIb alters receptor expression and associates with autoimmunity. I. Regulatory FCGR2B polymorphisms and their association with systemic lupus erythematosus.** *J Immunol* 2004, **172:** 7186-7191.

13. Blank MC, Stefanescu RN, Masuda E, Marti F, King PD, Redecha PB *et al*.: **Decreased transcription of the human FCGR2B gene mediated by the -343 G/C promoter polymorphism and association with systemic lupus erythematosus.** *Hum Genet* 2005, **117:** 220-227.

14. Chen JY, Wang CM, Ma CC, Luo SF, Edberg JC, Kimberly RP *et al*.: **Association of a transmembrane polymorphism of Fcgamma receptor IIb (FCGR2B) with systemic lupus erythematosus in Taiwanese patients.** *Arthritis Rheum* 2006, **54:** 3908-3917.

15. Li X, Wu J, Carter RH, Edberg JC, Su K, Cooper GS *et al*.: **A novel polymorphism in the Fcgamma receptor IIB (CD32B) transmembrane region alters receptor signaling.** *Arthritis Rheum* 2003, **48:** 3242-3252.

16. Floto RA, Clatworthy MR, Heilbronn KR, Rosner DR, MacAry PA, Rankin A *et al*.: **Loss of function of a lupus-associated FcgammaRIIb polymorphism through exclusion from lipid rafts.** *Nat Med* 2005, **11:** 1056-1058.

17. Kono H, Kyogoku C, Suzuki T, Tsuchiya N, Honda H, Yamamoto K *et al*.: **FcgammaRIIB Ile232Thr transmembrane polymorphism associated with human systemic lupus erythematosus decreases affinity to lipid rafts and attenuates inhibitory effects on B cell receptor signaling.** *Hum Mol Genet* 2005, **14:** 2881-2892.

18. Hitomi Y, Tsuchiya N, Kawasaki A, Ohashi J, Suzuki T, Kyogoku C *et al*.: **CD72 polymorphisms associated with alternative splicing modify susceptibility to human systemic lupus erythematosus through epistatic interaction with FCGR2B.** *Hum Mol Genet* 2004, **13:** 2907-2917.

19. van Mirre E, Breunis WB, Geissler J, Hack CE, de Boer M, Roos D *et al*.: **Neutrophil responsiveness to IgG, as determined by fixed ratios of mRNA levels for activating and inhibitory FcgammaRII (CD32), is stable over time and unaffected by cytokines.** *Blood* 2006, **108:** 584-590.

20. Pant PV, Tao H, Beilharz EJ, Ballinger DG, Cox DR, Frazer KA: **Analysis of allelic differential expression in human white blood cells.** *Genome Res* 2006, **16:** 331-339.
